# Small RNAs from *Bemisia tabaci* Are Transferred to *Solanum lycopersicum* Phloem during Feeding

**DOI:** 10.3389/fpls.2016.01759

**Published:** 2016-11-24

**Authors:** Paula J. M. van Kleeff, Marc Galland, Robert C. Schuurink, Petra M. Bleeker

**Affiliations:** Department of Plant Physiology, Swammerdam Institute for Life Sciences, University of AmsterdamAmsterdam, Netherlands

**Keywords:** small-RNA, whitefly, RNAseq, phloem, tomato

## Abstract

The phloem-feeding whitefly *Bemisia tabaci* is a serious pest to a broad range of host plants, including many economically important crops such as tomato. These insects serve as a vector for various devastating plant viruses. It is known that whiteflies are capable of manipulating host-defense responses, potentially mediated by effector molecules in the whitefly saliva. We hypothesized that, beside putative effector proteins, small RNAs (sRNA) are delivered by *B. tabaci* into the phloem, where they may play a role in manipulating host plant defenses. There is already evidence to suggest that sRNAs can mediate the host-pathogen dialogue. It has been shown that *Botrytis cinerea*, the causal agent of gray mold disease, takes advantage of the plant sRNA machinery to selectively silence host genes involved in defense signaling. Here we identified sRNAs originating from *B. tabaci* in the phloem of tomato plants on which they are feeding. sRNAs were isolated and sequenced from tomato phloem of whitefly-infested and control plants as well as from the nymphs themselves, control leaflets, and from the infested leaflets. Using stem-loop RT-PCR, three whitefly sRNAs have been verified to be present in whitefly-infested leaflets that were also present in the whitefly-infested phloem sample. Our results show that whitefly sRNAs are indeed present in tomato tissues upon feeding, and they appear to be mobile in the phloem. Their role in the host-insect interaction can now be investigated.

## Introduction

*Bemisia tabaci* (Hemiptera), commonly known as whitefly, is a polyphagous insect that is a threat for many crops across the globe. These insects can reduce crop yield in a number of ways; (1) through transmission of yield-limiting plant viruses (Navas-Castillo et al., 2011); (2) via honeydew excrement, which results in growth of sooty molds leading to a reduction of photosynthesis (Walling, 2008) or the release of the glycoside of salicylic acid (VanDoorn et al., 2015) or; (3) ingestion of phloem sap thereby depleting plants of photosynthetic compounds (Buntin et al., 1993).

Plants can defend themselves against herbivores and pathogens in various ways e.g., via physical barriers, volatile or non-volatile compounds, and through induction of defense responses controlled by various phytohormones (Walling, 2008; Kant et al., 2015). Trichomes can act both as physical barriers and as metabolite production facilities. Trichomes on the leaves will hinder small herbivores in their movement and finding suitable feeding places (Simmons and Gurr, 2005). In addition, trichomes can produce specialized metabolites such as repellent volatiles or exudates that can be toxic or that trap herbivores (Simmons and Gurr, 2005; Walling, 2008; Bleeker et al., 2009). The phytohormones involved in herbivore-defense responses are predominantly jasmonic acid (JA) and salicylic acid (SA). SA can antagonize the JA-mediated signaling responses (Koornneef and Pieterse, 2008). Adult whiteflies feeding on tomato induce the SA-response thereby suppressing the JA-response (Shi et al., 2014). During the feeding of whitefly nymphs on Arabidopsis, transcript levels of SA-induced genes became higher while JA-related transcript levels decreased (Kempema et al., 2007; Zarate et al., 2007).

After hatching from the egg, whitefly nymphs are mobile and will select the site where they will feed and develop into an adult while being immobile. Feeding is initiated by insertion of a specialized mouthpiece (stylet) through the leaf surface toward the phloem sieve elements in a mostly intercellular fashion (Pollard, 1955; Jiang et al., 1999; Jiang and Walker, 2003). This insertion is facilitated by the excretion of gel-like saliva, in a similar way as an aphid, and other stylet- and phloem-feeding insects (Jiang et al., 1999; Moreno et al., 2011). After the stylet enters the sieve element, watery saliva is excreted and ingestion of phloem sap starts (Jiang et al., 1999; Jiang and Walker, 2003). Plants try to close the opening made by the stylet by depositing callose and proteins (Kempema et al., 2007) and phloem-feeding insects try to counteract this (Will et al., 2007).

There is evidence that herbivore saliva contains factors that can manipulate plant defenses (Will et al., 2013; Sharma et al., 2014; Su et al., 2015; Peng et al., 2016; Villarroel et al., 2016). For hemipterans most knowledge stems from work with aphids: several salivary proteins (effectors) have been identified that affect aphid reproductive rate (Bos et al., 2010; Pitino and Hogenhout, 2013). The aphid salivary proteins C002, Mp1, and Mp2 increase fecundity, while Mp10 and Mp42 reduce aphid fecundity (Bos et al., 2010; Pitino and Hogenhout, 2013). The production of effector proteins by aphids seems to be analogous to that of plant pathogens to establish disease. Such plant pathogens can interfere with the defense response of their host by secreting effectors that interact with host proteins and modulate these to their benefit.

Besides effector-protein interactions, small non-coding RNAs (sRNAs) between 21 and 24 nucleotides long (nts) have been shown to mediate interactions between hosts and pathogens (Knip et al., 2014; Baulcombe, 2015). Regarding plants, one of the best-studied examples is the *Botrytis cinerea* infection of Arabidopsis and tomato (Weiberg et al., 2013). After fungal infection, 73 sRNAs from Botrytis were found in infected leaves (Weiberg et al., 2013). These Botrytis sRNAs take advantage of the plant's own silencing machinery to mediate their action (i.e., targeting ARGONAUTE 1). Another example comes from the green peach aphid (*Myzus persicae*) that displays reduced fecundity on Arabidopsis mutants affected in their miRNA biogenesis pathway (i.e., Dicer-like1 *dcl1* and Argonaute1 *ago1*; Kettles et al., 2013). These results indicate that sRNA pathways are not only involved in plant resistance against a phloem-feeding insect, but also suggest that aphids produce sRNAs that can influence plant-defense responses (Kettles et al., 2013).

Here we show that whiteflies transfer sRNAs to the host plant they are feeding from. To detect whitefly-specific sRNAs within the phloem of tomato plants small RNA sequencing was utilized. These sRNAs are detected in isolated phloem sap indicating they are mobile. The presence of three whitefly sRNAs in tomato was confirmed by means of stem-loop RT-PCR. Our findings are, to our knowledge, the first confirmation of the transfer of insect sRNA to phloem.

## Methods and materials

### Whitefly rearing and tomato infestation

Whiteflies (*B. tabaci* biotype B) were reared in a climatised chamber (Snijders, Tilburg; 28°C, 16 h light 150 μE m^−2^ s^−1^, RH 75%) as previously described (Bleeker et al., 2011), on a diet of cucumber plants (*Cucumis sativus*, Ventura, RijkZwaan). Two weeks after sowing, 5 tomato plants (*Solanum lycopersicum*, cultivar Moneymaker) were placed in a netted insect dome (60 × 60 × 90 cm) and infested with ±200 adult whiteflies (greenhouse 22–25°C, 16/8 h photoperiod at 500 μE m^−2^ s^−1^). Aiming for a consistent treatment with the different instar stages present, whiteflies (±100) were added 3 times per week until week 4 after sowing. In week 6 after sowing the samples for small RNA sequencing were collected.

### Phloem, nymph, and leaf collection for sRNA-seq

For phloem collection plants were kept in the greenhouse under standard greenhouse conditions (22–25°C, 16/8 h photoperiod at 500 μE m^−2^ s^−1^). Phloem sap was collected from control and whitefly-infested leaflets using the “EDTA” method (King and Zeevaart, 1974) during the light-period (see also Figure S1 and Tetyuk et al., 2013). Adult whiteflies were removed from treated leaflets by aspiration. Leaflets with a high density of nymphs were excised and the petioles were carefully submerged in phloem collection buffer (5 mM EDTA, 5 mM phosphate buffer pH 6.8). The petioles of 3–6 leaflets were then cut once more while submerged in buffer and placed in a 2-ml Eppendorf vial containing phloem-collection buffer to bleed for 30 min under high humidity. After this the leaflets were transferred to collection tubes with fresh phloem-collection buffer supplemented with protease inhibitor (1 Complete Protease Inhibitor Tablet (Roche) 100 ml^−1^ water) and phloem samples were collected for 6 h under high humidity before being snap frozen in liquid nitrogen. Nymphs (1st, 2nd, and 3rd instar) were collected from a total of 4 infested leaflet using an insect pin, pooled, and transferred to 100% acetone. For the infested sample (LW) in addition to nymphs, eggs were removed as accurate as possible as well, after which the leaflet samples were separately snap frozen in liquid nitrogen (*n* = 4). Untreated control leaflets (LC) were harvested in exactly the same way and at the same time point (*n* = 4). For an additional control, leaflets with the eggs remaining were included (LE, *n* = 4). For this adult whiteflies were placed on leaflets for 24 h after which the adults were removed.

### Total and small RNA isolation

Total RNA from phloem samples was isolated using concentrated TRIzol reagent (Life Technologies). The leaf samples (for each treatment four replicates pooled) and one nymph sample were ground in liquid nitrogen. Total RNA was isolated using the E.Z.N.A.® MicroElute RNA Clean Up Kit (Omega Bio-Tek). Briefly, TRIzol Reagent (Life Technologies) and chloroform was added according to the manufacturer's instructions. After centrifugation, the RNA-containing aqueous phase was collected, mixed with 1.5 volume of 100% ethanol and applied to a MicroElute spin column (Omega Bio-Tek). The column was washed according to the manufacturers's instructions: once with RWT buffer (Qiagen), once with RPE washing buffer (Qiagen) and finally with 80% ethanol. The RNA concentration was measured on a NanoDrop ND-2000 (Thermo Scientific) and RNA integrity was examined using the 2200 TapeStation System with Agilent RNA ScreenTapes (Agilent Technologies).

Total RNA was spiked with ERCCs spike-in mix 1 (Life Technologies) as well as a synthetic spike-in set for Size Range Quality Control (SRQC) together with an External Reference for Data Normalization (ERDN; Locati et al., 2015). Both phloem samples from the control and whitefly-infested plants were not spiked. The total RNA was divided in a large and a small fraction. The large RNA fraction was bound to a mirVana™ spin column (mirVana™ miRNA Isolation Kit, Life Technologies) according to the manufacturer's instructions. Small RNAs (<200 nts) were purified from the flow-through by adding ethanol to a final concentration of 65% (v/v) and bound to an E.Z.N.A.® MicroElute spin column. The column was washed once with RWT buffer, once with RPE buffer and once with 80% ethanol (Qiagen). The concentration and integrity of small RNA was examined as described above.

### Next-generation sequencing

Bar-coded small RNA libraries of the 6 different samples were generated according to the manufacturer's protocols using the Ion Total RNA-Seq Kit v2 and the Ion Xpress™ RNA-Seq bar-coding kit (Life Technologies). The size distribution and yield of the bar-coded libraries were assessed using the 2200 TapeStation System with Agilent D1K ScreenTapes (Agilent Technologies). Sequencing templates were prepared on the Ion Chef™ System using the Ion PI Hi-Q Chef Kit (Life Technologies). Sequencing was performed on an Ion Proton™ System using Ion PI v3 chips (Life Technologies) according to the manufacturer's instructions.

### Bioinformatic analyses

Bioinformatic analyses were done using the Snakemake workflow management tool (Köster and Rahmann, 2012) to generate bioinformatic pipelines. Software used was Bowtie2 v2.1.0 (Langmead and Salzberg, 2012), Samtools v1.2 (Li et al., 2009), Python v3.3.3, Python package Pandas 0.14.1, and Biopython 1.64 (Cock et al., 2009), STAR v2.4.0 (Dobin et al., 2013), R v3.2.1 (R Core Team, 2016). All sequences <18 and >40 nts were removed. Contaminating sequences were removed by alignment to plant virus databases (Adams and Antoniw, 2006), other types of RNA (rRNA, tRNA, snoRNA, degraded messenger RNA, mitochondrial RNA) using the RFAM 12.0 database for tomatoes (excluding microRNA; Nawrocki et al., 2015) and the publically available tomato transcriptome [ITAG2.3, solgenomics.net Tomato Genome Consortium, 2012]. sRNAs were normalized for comparisons and expressed as RPKM (Reads Per Kilobase per Million mapped reads; Table S1).

The online psRNATarget tool (Dai and Zhao, 2011) was used to retrieve mRNA targets of selected miRNAs (using the *Solanum lycopersicum* ITAG2.4 cDNA reference) with default parameters: a seed region length of 20 nts to score complementarity between target and miRNA and a target accessibility (maximum energy required to open the mRNA secondary structure around the target site) of 25.

### Stem-loop RT-PCR of small RNAs

Stem-loop RT-PCR of small RNAs was conducted to confirm findings of the sRNA-seq. For this analyses total RNA was isolated from four biological replicates of leaflet samples using the method described above and primers for the specific small RNAs were designed (Varkonyi-Gasic et al., 2007; Kramer, 2011; Table S2). A total of 100 ng RNA per sample was used for a reverse transcriptase reaction (RevertAid H Minus reverse Transcriptase, Thermo fisher) in a total volume of 20 μL with small RNA specific RT-primers (Table S2). A pulse reverse-transcriptase reaction (RT) was used (Varkonyi-Gasic et al., 2007). For the stem-loop RT-PCRs, 2 μL of RT reaction was used as template in a total volume of 50 μL for 35 cycles with an annealing temperature of 58°C. PCR products were cloned with CloneJET PCR cloning kit (Thermo scientific) and sequenced to validate amplicon specificity.

## Results

### Small RNA sequencing: detection of small RNAs in phloem and leaves

**Figure 1:**
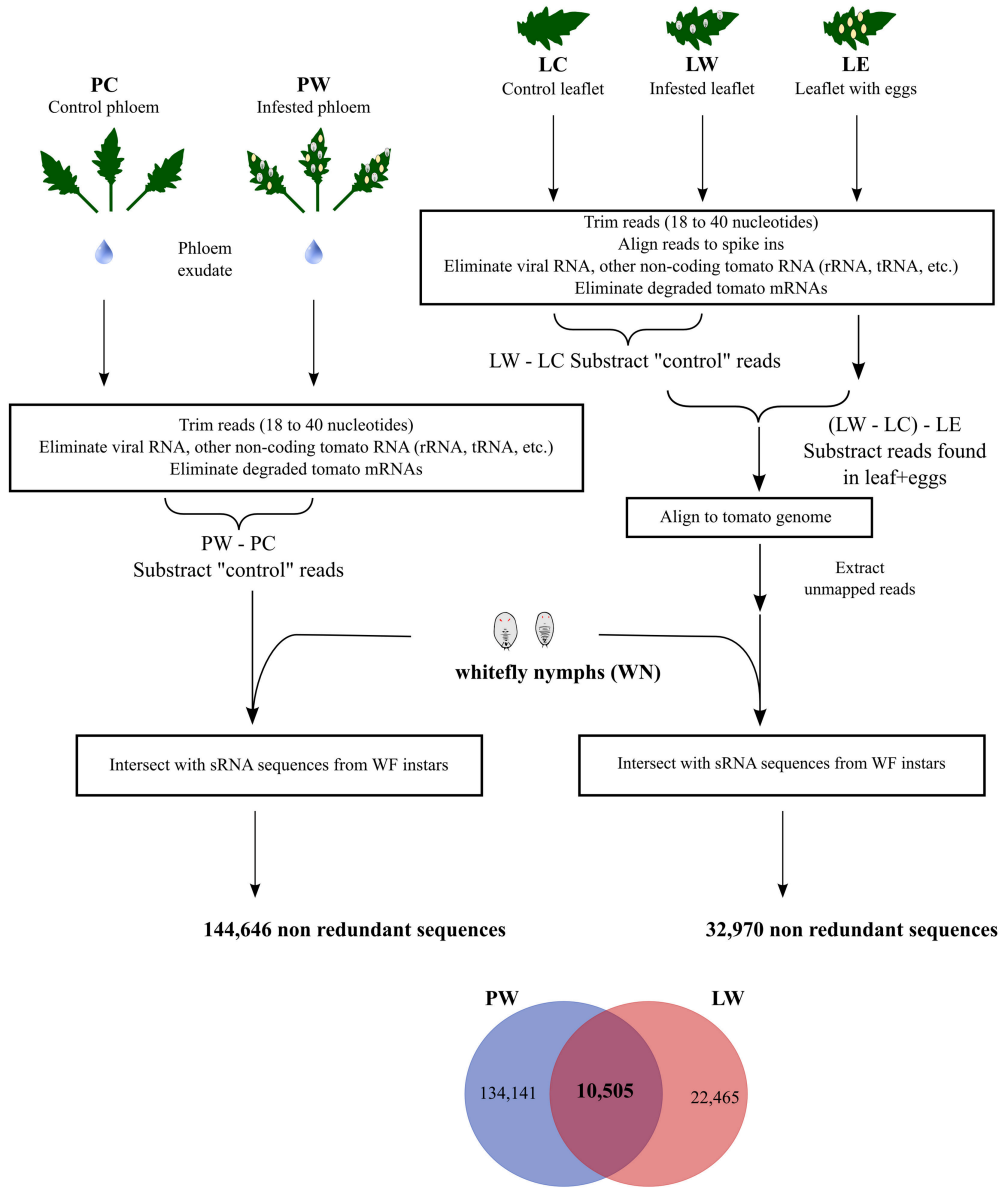
**Bioinformatic pipeline**. Small RNAs (sRNAs) were isolated from tomato-phloem exudates from either control leaflets (PC) or leaflets infested with whitefly nymphs (PW), and from control tomato leaflets (LC), whitefly nymphs (WN), leaflets first infested with whiteflies but with adults and nymphs subsequently removed (LW) and tomato leaflets with only eggs (LE). A bioinformatic workflow (see main text for details) was implemented yielding 10,505 non-redundant small RNAs present in feeding whitefly nymphs plus identified in both infested tomato phloem and leaflets.

Phloem samples were collected from uninfested tomato leaflets (Phloem control, PC) and compared to phloem samples from tomato leaflets infested with whitefly nymphs and eggs (Phloem whitefly, PW, Figure 1). Whitefly nymphs (WN) were separately collected from leaflets of the same plants used to obtain phloem sap exudates (Figure 1). After sRNA sequencing we obtained 31,231,948 sRNAs in the WN sample and 5,370,176 and 7,321,768 sRNAs in the PC and PW samples respectively (see also Table S1). To determine which sRNAs originated from whiteflies and were present in phloem, a bioinformatic pipeline was designed (Figure 1). For this analysis, sequences shorter than 18 nucleotides (nts) and longer than 40 nts were removed. Next, we removed sequences that aligned to plant viruses (Adams and Antoniw, 2006), other types of RNA (rRNA, tRNA, snoRNA, degraded messenger RNA, mitochondrial RNA) using the RFAM 12.0 database for tomatoes (excluding microRNA; Nawrocki et al., 2015) and the publically available tomato transcriptome (ITAG2.3, Tomato Genome Consortium, 2012). To eliminate sequences present in PW but unrelated to whitefly infestation, PC sRNAs were excluded from the PW sRNAs (PW–PC, Figure 1). Of these PW-PC sRNAs a final 144,646 non-redundant sequences overlapped with WN sequence (Figure 1, Table S1) and were regarded as putatively transferred from the insect into the phloem.

In order to further validate whitefly-specific sRNAs present in tomato, we additionally sequenced sRNAs isolated from whole tomato leaflets (Figure 1, Table S1). We obtained 36,793,380 sRNAs from uninfested tomato leaflets (Leaf Control, LC), 33,780,469 sRNAs from infested leaflets after removal of nymphs (Leaf whitefly, LW) and 32,730,583 sRNAs from leaflets with only eggs (Leaf Eggs, LE). LC sRNAs were subtracted from the LW sRNAs (LW–LC, Figure 1). Next, to correct for any whitefly-specific egg sRNA that could have been left on the leaf surface of LW, the LE sequences were removed (Figure 1). The remaining whitefly nymph sRNAs were subsequently aligned against the tomato genome and the unmapped sequences were aligned with the tomato-fed WN sequences to find nymph-specific sRNAs. By doing so, we ended up with 32,970 non-redundant sequences (Figure 1). Finally, we searched (qualitatively) for sRNAs that would be both present amongst the 144,464 sRNAs coming from PW sample and the 32,970 sRNAs coming from infested LW sample, and found in WN nymph sample. This resulted in 10,505 non-redundant (Figure 1, Table S1) putative whitefly sRNAs found in both phloem exudate and leaflets containing phloem of whitefly-infested plants.

### Length distribution

**Figure 2:**
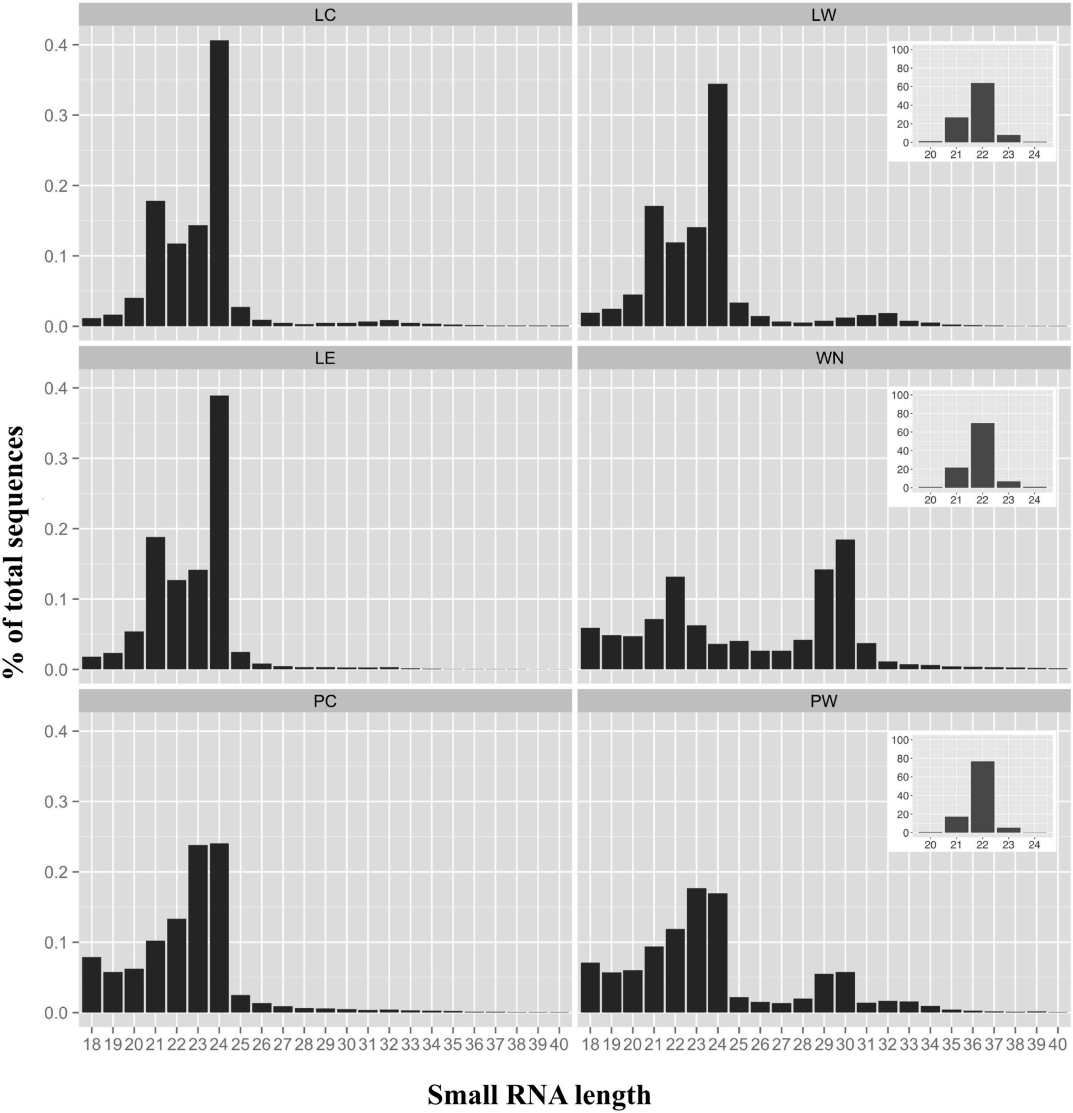
**Size distribution of sRNAs**. Size distributions and percentages of small RNAs (sRNAs) between 18 and 40 nucleotides are indicated for each sample. LC, control leaflets (not infested with whitefly nymphs); LW, leaflets first infested with whiteflies but with adults and nymphs subsequently removed; LE, leaflets with whitefly eggs; WN, whitefly nymphs; PC, phloem control (not infested); PW, phloem infested with whitefly nymphs. Insets for LW, WN, and PW represent the size distributions and percentages of the sRNAs matching previously described whitefly miRNAs (Guo et al., 2013).

The sRNA-length distributions from the different libraries ranging from 18 to 40 nts are shown in Figure 2. The sRNA length distributions of the three leaf samples (LC, LW, and LE) were very comparable with an expected major peak at 24 nts (35–40% of all sequences) and a minor peak at 21 nts (Figure 2). Compared to tomato leaf samples, both phloem samples exhibited a slightly different length distribution with a peak at 23–24 nts (~18–25% of all sequences). However, the sRNA length distribution of the whitefly nymphs (WN) was distinctly different from the other samples with two major peaks; one at 22 nts (~12% of all sequences) and one at 29–30 nts (~15–20% of all sequences; Figure 2). The 29–30 nt sequences, apparent in the nymph sample, appeared to be a well-defined peak in the phloem sample from the nymph-infested leaflet (PW), compared to the control phloem (PC).

Next, the sRNAs from whitefly-infested samples LW, WN, and PW were compared to 185 whitefly miRNA sequences found in two different biotypes of *B. tabaci* (Guo et al., 2013). All previously described whitefly miRNAs had a length comprised between 20 and 24 nts (Guo et al., 2013). Accordingly, 150 sRNAs of our WN sample could be exactly matched to the whitefly miRNAs published earlier (Table S3) and had a length predominantly centered around 22 nts, a feature characteristic of insect miRNAs (Figure 2, insets). The most abundant miRNAs found in the WN sample were miR-276a, miR-317 and miR-14 that appear to be conserved as well in other insects e.g., *Bombyx mori, Apis mellifera, Drosophila melanogaster* (Yin et al., 2016; Table S3).

### Confirmation of small RNAs in leaf samples

**Figure 3:**
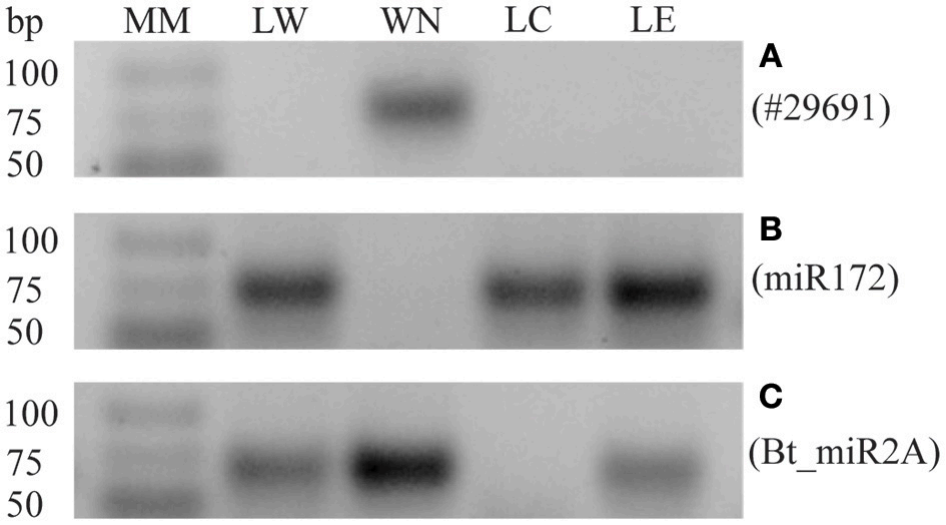
**Quality check of leaflet samples by stem-loop RT-PCRs. (A)** Expression of nymph-specific sRNA #29691 in: leaflet first infested with whiteflies but with adults and nymphs subsequently removed (LW), whitefly nymphs (WN), control leaflet (LC), or leaflet with eggs (LE), showing a specific band in WN only. **(B)** Validation of the expression of tomato specific sRNA Sly_miR172 in the different samples. Expression is detected in LW, LC, and LE, but not in the WN sample. **(C)** Expression of the known whitefly-specific miRNA Bta_miR2A is also detected in LE. Similar results were obtained in four biological replicates. Molecular Mass (MM), GeneRuler Ultra Low Range DNA ladder (Thermo Scientific).

A sensitive stem-loop RT-PCR (Varkonyi-Gasic et al., 2007) was used to confirm the presence or absence of whitefly sRNAs in four biological replicates of whitefly-infested tomato leaflets (LW), non-infested leaflets (LC), leaflets with eggs (LE), and the *B. tabaci* nymph sample (WN). To verify there was no sRNA from whitefly nymph contaminating the LW sRNAs other than those transferred by the whitefly, a 29-nt sRNA (# 29691) was amplified as this sRNA proved particularly abundant in the WN sample. Figure 3A shows that sRNA #29691 was indeed specific to the nymph sample and was absent in the LW, LC, or LE samples, indicating that there is no whitefly nymph contamination in the LW sample after the infestation and that nymphs had been successfully removed. Sample quality was further checked using a known *B. tabaci* specific sRNA (Bta_miR2A; Guo et al., 2013) and a known tomato miR172 that is conserved among land plants (Taylor et al., 2014). Accordingly, the plant-specific miR172 was detected in all leaf samples (Figure 3B) while the whitefly Bta_miR2A was detected in the nymph samples of *B. tabaci* reared on tomato and in the leaf samples containing eggs (LW and LE; Figure 3C).

### Whitefly small RNAs transferred to tomato

**Figure 4:**
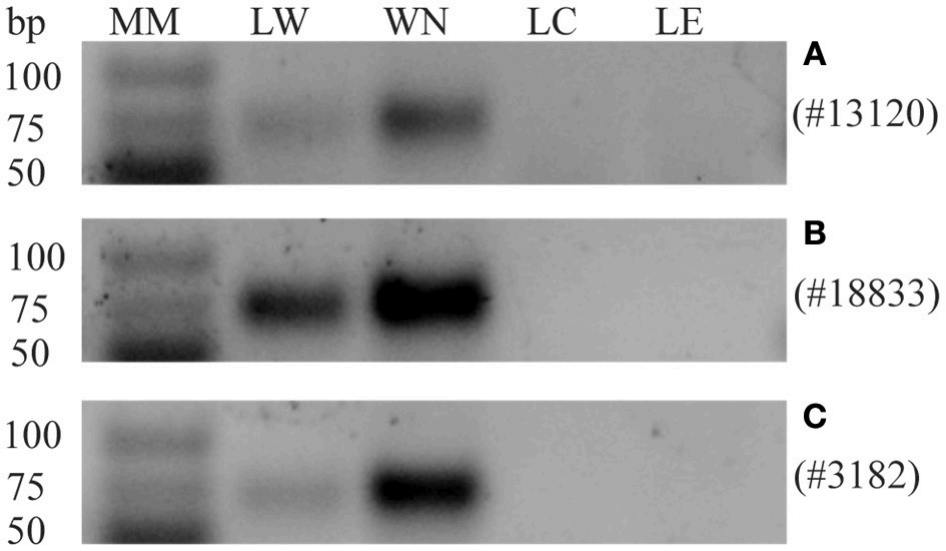
**Detection of candidate transferred whitefly sRNAs in the nymph and leaflet samples by stem-loop RT-PCRs**. Expression of sRNAs in: leaflet first infested with whiteflies but with adults and nymphs subsequently removed (LW), whitefly nymphs (WN), control leaflet (LC) or leaflet with eggs (LE). **(A)** sRNA #13120, a specific band is detected in LW and WN but not in LC and LE. **(B)** #18833, a specific band is detected in LW and WN but not in LC and LE. Similar results for #13120 and #18833 were obtained in four biological replicates. **(C)** #3182, a specific band is detected in LW and WN but not in LC and LE in three out of four replicates. One biological replicate showed also a band in LC and LE. Molecular Mass (MM), GeneRuler Ultra Low Range DNA ladder (Thermo Scientific).

sRNA sequencing of the phloem samples identified the presence of mobile whitefly-originating sRNAs. We selected three whitefly candidate sRNAs from the final list (Table 1) for validation using stem-loop PCR on leaflets. The criteria for selecting these specific candidates from the sRNAseq data were (1) a length between 23 and 24 nt (Figure 2), (2) present among highest counts in nymphs, (3) presence in whitefly-infested leaflets (LW), in phloem from leaflets infested with whiteflies (PW) and in the *B. tabaci* nymph (WN) sample, while absent in the control leaflet (LC), absent in leaflets with only eggs (LE), and absent in the phloem control sample, and finally (4) preferably matching an insect-like or an unknown small RNA in the miRBase. From the three selected sRNAs, sRNA #13120, and #18833 were annotated as insect miR305 and miR1175-3p, respectively, using the miRBase (Kozomara and Griffiths-Jones, 2014). sRNAs #13120 (Figure 4A) and #18833 (Figure 4B) were present in nymph and were found back in three out of four LW samples while being absent in all LC and LE samples. sRNA #3182 did not provide a match in the miRBase but could be amplified in nymph and all infested leaf samples, however it was found in one out of four control samples. Overall, whitefly sRNAs could be detected within the leaflet samples (Figure 4C) on which nymph feeding took place. Since two out of three candidate small RNAs were found exclusively in the infested samples PW and LW, we conclude that whiteflies transfer small RNAs to the phloem, which then have the potential to move.

**Table 1:** **List of selected putative whitefly sRNA candidates with normalized counts (RPKM)**.

**Id**	**sRNA sequence (5′–3′)**	**Counts**	**Length**	**Best miRBase21 homolog**
–	ACCGGCGGCGCGGUGAGGCACC	44	22	Unknown
–	CACCGGCGGCGCGGUGAGGCACC	47	23	Unknown
–	CACCGGAAGGAUUGACAGAUU	66	21	*Acyrthosiphon pisum* miR-263b
–	UGAGAUUCAACUCCUCCAUCUUAU	1574	24	*Bombyx mori* miR-1175
–	AGCAGAGUGGCGCAGUGGAAGC	386	22	*Monodelphis domestica* miR-885
3182	**UAGUAGCUAACGACGAUUCCUUU**	**957**	**23**	**NA**
–	UAAGGCACGCGGUGAAUGCCAUU	1105	23	*Panagrellus redivivus* miR-124
–	UGGUAACUCCACACCACCGUUGGC	1713	24	*Acyrthosiphon pisum* miR-2765
–	GCGGGUGUCGGCGGCCGUG	52	19	*Pongo pygmaeus* miR-118
–	UGAGAUCAUCGUGAAAGCUGAUA	543	23	*Apis mellifera* bantam stem-loop
–	CAAGCUCGUUGAAGUAUACCCAU	531	23	*Petromyzon marinus* miR-133a
–	UAAGUACUCCGUGCCGCAGGA	899	21	*Daphnia pulex* miR-252a
–	UCAGGCGGGCAAUCGCCGGG	157	20	*Ectocarpus siliculosus* miR3453
–	UCGCGGGUGUCGGCGGCCGUGAGC	31	24	*Pongo pygmaeus* miR-1181
–	GGCGGCAAUCGCCGGGGCCCU	9	21	*Mus musculus* miR-3104
–	UGGACGGAGAACUGAUAAGGGCU	553	23	*Drosophila melanogaster* miR-184
–	AUACAGGGGAGUAAGGGUUUGU	316	22	*Monodelphis domestica* miR-7398j
18833	**UGAGAUUCAACUCCUCCAUCUUA**	**1166**	**23**	***Bombyx mori*** **miR-1175**
–	GAAGGCCCUACAACGCGGACCCC	1557	23	*Equus caballus* miR-1905a
–	UAUCACAGCCAUUUUGACGUGCCU	1037	24	*Drosophila melanogaster* miR-13b-1
13120	**AUUGUACUUCAUCAGGUGCUCUGU**	**1275**	**24**	***Drosophila melanogaster*** **miR-305**
–	UUAAAAAGUGAUUUCACCACGG	750	22	*Ornithorhynchus anatinus* miR-1334

*The sRNAs indicated in bold were detected with stem-loop RT-PCR*.

### Prediction of whitefly sRNAs targets in tomato

To get insight into putative roles of these three whitefly sRNAs, the online psRNAtarget tool was used (Dai and Zhao, 2011) to predict putative tomato mRNA targets. By doing so, putative targets for the sRNAs #13120 and #18833 were found. sRNA #13120 is predicted to target four different exocyst complex proteins of which two are expressed in tomato leaves (Table 2). Another interesting putative targets of sRNA #18833 are three closely related xylanase inhibitors (Table 2). They are moderately similar (between 56 and 58% identity at the amino acid level) to a previously described tomato xyloglucan-specific endoglucanase inhibitor (Qin et al., 2003).

**Table 2:** **List of predicted mRNA targets from the selected whitefly small RNA found in tomato tissues**.

**id**	**sRNA sequence (5′–3′)**	**Length**	**mRNA target**	**Annotation**
13120	AUUGUACUUCAUCAGGUGCUCUGU	24	Solyc09g075400.2.1	Putative DNA-ligase
13120	AUUGUACUUCAUCAGGUGCUCUGU	24	Solyc10g019140.1.1	Exocyst complex component protein
13120	AUUGUACUUCAUCAGGUGCUCUGU	24	Solyc10g019110.1.1	Exocyst complex component protein
13120	AUUGUACUUCAUCAGGUGCUCUGU	24	Solyc11g050710.1.1	Exocyst complex component protein
13120	AUUGUACUUCAUCAGGUGCUCUGU	24	Solyc03g095410.2.1	Exocyst complex component protein
13120	AUUGUACUUCAUCAGGUGCUCUGU	24	Solyc02g085940.2.1	Unknown protein
13120	AUUGUACUUCAUCAGGUGCUCUGU	24	Solyc08g062170.1.1	Unknown protein
13120	AUUGUACUUCAUCAGGUGCUCUGU	24	Solyc01g009030.2.1	ATP synthase regulation protein
13120	AUUGUACUUCAUCAGGUGCUCUGU	24	Solyc01g111270.2.1	Armadillo protein
18833	UGAGAUUCAACUCCUCCAUCUUA	23	Solyc01g079980.2.1	Xylanase inhibitor
18833	UGAGAUUCAACUCCUCCAUCUUA	23	Solyc01g079960.2.1	Xylanase inhibitor
18833	UGAGAUUCAACUCCUCCAUCUUA	23	Solyc03g082470.2.1	Leucine-rich repeat receptor-like protein kinase
18833	UGAGAUUCAACUCCUCCAUCUUA	23	Solyc02g084980.2.1	Galactinol synthase
18833	UGAGAUUCAACUCCUCCAUCUUA	23	Solyc11g008350.1.1	Kinesin-like protein
18833	UGAGAUUCAACUCCUCCAUCUUA	23	Solyc01g079970.2.1	Xylanase inhibitor
18833	UGAGAUUCAACUCCUCCAUCUUA	23	Solyc02g037490.1.1	Acyl-activating enzyme
18833	UGAGAUUCAACUCCUCCAUCUUA	23	Solyc00g005160.1.1	Retrovirus-related Pol polyprotein from TNT transposon
3182	UAGUAGCUAACGACGAUUCCUUU	23	Solyc02g085990.1.1	Unknown protein

## Discussion

### Cross-kingdom interactions mediated by sRNAs

Besides ingesting plant sap, phloem-feeding insects such as aphids manipulate plant defenses by secreting protein effectors that can improve host colonization and reproductive fitness (Louis and Shah, 2013). However, the precise molecular mode of action of aphid effectors remains elusive. Along with an array of notorious plant-viruses (Rosen et al., 2015), whiteflies theoretically could also transfer effector proteins into the phloem while feeding. Here we show that *B. tabaci* also appears to transfer sRNAs into the tomato phloem. Transfer of sRNAs from whiteflies could putatively be involved in transcriptional or post-transcriptional gene silencing inside the host and form an additional way for the insect to manipulate host defenses. There is increasing experimental evidence that sRNAs can mediate cross-kingdom interactions between plant and microorganisms (Knip et al., 2014), with perhaps the most convincing examples being those of Botrytis-Arabidopsis and Botrytis-tomato (Weiberg et al., 2013). One of the major difficulties in the field is to establish with certainty that a specific sRNA has been transferred by the “invader organism” into the host rather than being produced by the attacked host. Genome availability of *B. cinerea*, tomato and *Arabidopsis thaliana* was a major advantage that led to the discovery that sRNA can promote fungal pathogenicity (Weiberg et al., 2013). We did not have a draft or complete *B. tabaci* genome sequence to our disposal and this work therefore relied on a bioinformatic pipeline to search for whitefly sRNAs present in the phloem of whitefly-infested tomato (Figure 1). To further verify the sequences found in the tomato phloem, the sRNAs present only in the PW phloem were compared to the sRNAs of the whitefly nymph (WN) sample. Additionally, they were cross-referenced to sRNAs from whitefly-infested leaflets (LW) after elimination of non-infected leaves (LC) and leaflet with eggs (LE), in case not all eggs were removed from the LW leaflet surface. sRNAs were subsequently aligned to the tomato genome to remove tomato sRNAs especially from repetitive regions (Figure 1), leaving us with potentially 10,505 whitefly-specific sRNAs present in plant tissue. The final LW sample (nymph sRNAs in LW–LC–LE) contains less phloem than phloem exudate itself (nymph sRNAs in PW–PC) as the starting material contains many other tomato cell types that have been consequently filtered out. The remaining 22,465 sequences that do not match the phloem sequences can contain e.g., whitefly sRNAs from phloem companion cells. The phloem exudate samples are more concentrated and thus contain more (134,141) putative whitefly sRNAs than the leaflet samples.

A great diversity of sRNAs has been found in the phloem sap of several species including pumpkin, cucumber, lupin, and Arabidopsis, in the absence of major pathogen infection or pest infestation (Yoo et al., 2004). These phloem sRNAs typically had a length between 18 and 25 nts with a major peak at 23 nts (Yoo et al., 2004), which is consistent with the sRNA length distribution found in our non-infested control samples (LC and PC, Figure 2). In leaves, a major peak at 24 nts is very common (see, Itaya et al., 2008 for an example in tomato), which has long been associated with transcriptional gene silencing especially of repetitive sequences e.g., transposons (Borges and Martienssen, 2015). In both phloem and leaflet samples, we found conserved miRNAs such as miR156 and miR172 known to act in concert to regulate flowering time (Spanudakis and Jackson, 2014) and miR159 previously identified in cucurbit phloem (Yoo et al., 2004). Finding such miRNAs among the most abundant phloem sRNAs is consistent with previous studies (Yoo et al., 2004; Rodriguez-Medina et al., 2011; Bhogale et al., 2014). We also observed Solanaceae-specific miR482 and miR6022 among the most abundant miRNAs in the non-infested phloem PC and LC sRNAs (Table S4). The collection of phloem was performed after an initial “bleed” period of 30 min to limit sample contamination by other types of cellular content. Nymphs were feeding on the leaflet at 2–3 cm distance from the petiole phloem collection site indicating mobile whitefly sRNAs in the plant phloem. It was estimated that contamination of phloem exudates by companion cell breakage composed around 2% of the exudate (Atkins et al., 2011). Thus, it is likely that the identified sRNAs in the phloem originate from the sieve elements although some contamination from neighboring cells cannot be completely ruled out.

The presence of whitefly nymphs on tomato leaflets caused 29–30 nts sRNAs to appear in the phloem of infested plants (Figure 2). These longer sRNAs were indeed also observed in the whitefly nymphs (Figure 2). A similar sRNA length distribution has previously been found in adult whiteflies (Guo et al., 2013; Wang et al., 2016) and similar sized sRNAs (between 26 and 30 nts) have been reported for other insects e.g., cotton-melon aphid (*Aphis gossypii*) or the brown planthopper (*Nilaparvata lugens*; Chen et al., 2012; Sattar et al., 2012). These longer sRNAs are assumed to be Piwi-interacting RNAs (piRNAs) and are known to be a large class of non-coding RNAs in animals, specifically linked to genome stability in germ-line cells and silence transposons (Vagin et al., 2006). In Drosophila (*D. melanogaster*), for example, piRNAs are produced in a Dicer-independent manner from transposon-rich genomic clusters and specifically silence transposon expression in the germline (Iwasaki et al., 2015). Sattar et al. (2012) found that sRNAs with a length between 26 and 27 were overrepresented in the cotton-melon aphid, *A. gossyppii*, when infesting melon plants containing the *Vat* aphid resistance gene. Similar to *B. tabaci*, there is no annotated genome for *A. gossypii* but in this case the authors could make use of an *A. pisum* transposon database to show that around 50% of these 26–27 sRNAs actually derived from transposons. Another ~5% matched from the primary endosymbionts in aphids, *Buchnera aphodicola* (Sattar et al., 2012). In this study, we cannot completely rule out that the 29–30 nts sRNA originate from tomato. Nevertheless, when trying to align the five most abundant 29 or 30 nts sRNA to the tomato Heinz genome sequence (Figure 1), no full-length alignments were found. Assuming these particular sRNAs are indeed piRNAs involved in insect germline development, it remains elusive as to if and how the enrichment in the phloem of whitefly-infested leaves (Figure 2) is biologically relevant.

### Insect salivary small RNAs transferred into host

Our bioinformatic pipeline identified whitefly sRNAs in tomato phloem of leaflets where nymphs were feeding. These sRNAs most likely found their way into the phloem via the whitefly saliva. During feeding whiteflies salivate into the phloem after which they ingests phloem sap (Figure S1; Pollard, 1955; Jiang et al., 1999; Jiang and Walker, 2003). Since whitefly nymphs are immobile and feed for long periods of time, one can expect to find components of whitefly saliva in the phloem. Salivary glands of phloem feeding insects like aphids and whitefly have been subjected to RNA sequencing and proteomics to obtain insight in the transcriptome and proteome (Carolan et al., 2011; Su et al., 2012; Rao et al., 2013). Also, aphid saliva has been collected and used for proteomics studies (Rao et al., 2013; Chaudhary et al., 2015). In the current study, phloem from whitefly-infested tomato was used, as obtaining salivary glands from adult whiteflies, though feasible (Ghanim et al., 2001; Su et al., 2012), proved too challenging in nymphs. In addition, the saliva composition of insects is not necessarily the same as the composition of the salivary gland, which includes cell membranes and ducts (Rao et al., 2013). Moreover, in order to collect sufficient saliva, it requires the culturing of large amounts of adult whiteflies for a prolonged period on an artificial diet (Su et al., 2015; VanDoorn et al., 2015), which was technically not feasible. Also, it has been reported that the composition of insect saliva differs when feeding on artificial diet and on different plant species (Habibi et al., 2001; Cooper et al., 2010). Finally, as nymphs are immobile while feeding for long periods of time it might increase the chances of actually identifying whitefly sRNAs in phloem.

To our knowledge, miRNAs have so far only been identified in the saliva of mosquito (*Aedes aegypti*; Maharaj et al., 2015). Interestingly, miRNAs closely related (one nucleotide difference) to our #13120 (Bta_miR305-pGtoU) and #18833 (Bta_miR1175-3p+A) were also found in the saliva of *A. aegypti*, particularly after sucrose feeding (Maharaj et al., 2015). Whether saliva secreted miRNAs are conserved among fluid-feeding insects remains to be seen. Since sRNAs of prokaryotes are generally bigger than 100 nt (Gottesman and Storz, 2011) and our cut-off for analysis was <40 nt, it is unlikely that the miRNAs presented here originate from symbionts present in the whitefly. Nevertheless, we aligned the 10,505 sRNAs to the genome of *Rickettsia* sp. Strain MEAM1 (Genbank AJWD00000000.2), and found no matches.

A possible source of contamination for the sRNA sequencing of leaflet samples could be part of nymphs still attached to the leaflet. However, in Figure 3A it was shown that nymph tissue was removed from leaflets or at least below the level of detection. The bands visible in the LW samples (Figures 4A–C) are therefore very unlikely to originate from nymph tissue still present on the leaflets. The plant specific sRNA miR172 was only found in leaf samples and not in the whitefly nymphs (Figure 3B) though this could have been possible since sRNAs have been found previously ingested by aphids (Sattar et al., 2012). Similarly, Bta_miR2A was detected only in the nymph and whitefly-infested samples (Figure 3C), showing that the miR2A of the eggs on leaflet (LE) samples was even detectable by stem-loop RT-PCR.

The three sRNAs investigated here were very likely transferred from whitefly into tomato. All three candidates were identified in whitefly-infested material and in the nymphs themselves. sRNA #13120 (Bta_miR305-pGtoU) (Figure 4A) and #18833 (Bta_miR1175-3p+A) (Figure 4B) have been detected in LW samples but not in the LC or LE sample. Family members of two of these candidates have been previously identified in whitefly (Guo et al., 2013). For the third candidate (#3182), no similarity was found with previously identified whitefly sRNAs or with other sequences in the miRBase. This sRNA has been detected in all of the replicates of whitefly-infested leaf samples and is very abundant in the nymph sample but could be detected once out of 4 in control leaflets. Despite the fact that it cannot be completely ruled out, it is highly unlikely that #3182 derives from tomato. Sly-miR172, a very abundant tomato miRNAs present in our phloem sample could not be detected in our nymph sample while #3182 was found in the insect in a relative high level.

### Small RNA as effectors?

Pathogens and insects are known to transfer proteins into host plant cells in order to suppress host immunity (Dangl et al., 2013; Will et al., 2013; Su et al., 2015). In fact, it has been postulated that phloem-feeding insects employ a suit of proteins that are passed from the saliva into the phloem during feeding which could act as effector proteins that suppress plant defenses. Besides proteins, non-protein salivary factors can act as an effector (Su et al., 2015). Here we describe, for the first time, the transfer of putative salivary non-coding sRNAs from whitefly and postulate that they might target tomato host proteins. Small RNAs could facilitate the interaction between organisms by improving the attackers chance of survival (Weiberg et al., 2013; Knip et al., 2014) or improve fecundity (Sattar et al., 2012). In plants, the high base complementarity between the sRNA and the target mRNA has been successfully used to predict post-transcriptional regulations by sRNAs (Ding et al., 2012). Further validation and characterization of these mRNA targets is currently under investigation.

## Author contributions

PvK and MG contributed equally to this paper and shared first authors. MG analyzed the RNAseq data and PvK did the wet-lab experiments. PB conceived the project and supervised MG. RS supervised PvK and contributed to discussions. All four authors wrote and carefully read and approved the final manuscript.

## Funding

This work was supported by funding from the European Union (H2020, DURETO project #655656) to MG and the NWO VIDI grant 12988 awarded to PB.

### Conflict of interest statement

The authors declare that the research was conducted in the absence of any commercial or financial relationships that could be construed as a potential conflict of interest. The reviewer HJ and handling Editor declared their shared affiliation, and the handling Editor states that the process nevertheless met the standards of a fair and objective review.
