# Supplementary material for: Small RNAs from Bemisia tabaci Are Transferred to Solanum lycopersicum Phloem during Feeding
Source: Front Plant Sci. 2016 Nov 24;7:1759. doi: 10.3389/fpls.2016.01759 (PMC5121246; doi:10.3389/fpls.2016.01759)
Supplement: Supplementary file 1 [file Table1.PDF]

Table S1. Number of sRNAs at each analytical step. PC: Phloem control, PW: phloem from whitefly infested plants, LC: leaf control, LW: leaf infested with whiteflies, LE: leaf with eggs deposited on, WN: whitefly nymphs

	Redundant sRNAs			Non-redundant sRNAs		
	PC	PW	WN	PC	PW	WN
Original	5,370,176	7,321,768	31,231,948	3,027,013	4,113,001	17,186,095
After trimming (18 nts < sRNA < 40 nts)	1,999,661	2,958,626	-	1,305,216	1,857,261	-
After alignment to plant virus db	1,990,582	2,947,567	-	1,299,654	1,850,598	-
After alignment to RFAM (rRNA, tRNA, snoRNA, etc.)	1,742,553	2,600,385	-	1,161,605	1,658,597	-
After alignment to tomato mRNA	1,664,670	2,512,089	-	1,112,412	1,603,489	-
PW-PC	1,897,658		-	319,030		-
PW-PC intersect WN	4,346,653			144,646 (set #1)		

	Redundant sRNAs				Non-redundant sRNAs			
	LC	LW	LE	WN	LC	LW	LE	WN
Original	36,793,380	33,780,469	32,730,583	31,231,948	21,956,804	19,461,107	19,516,546	17,186,095
After alignment to spike ins	36,045,154	32,985,853	32,033,206	-	21,560,612	19,054,313	19,154,986	-
After trimming (18 nts < sRNA < 40 nts)	31,394,680	25,383,394	26,107,175	-	18,905,543	15,018,386	15,897,282	-
After alignment to plant virus db	31,319,721	25,310,008	26,031,234	-	18,861,008	14,976,128	15,852,319	-
After alignment to RFAM (rRNA, tRNA, snoRNA, etc.)	29,015,282	21,596,689	24,123,438	-	17,659,020	13,056,740	14,842,019	-
After alignment to tomato mRNA	27,286,742	20,247,471	22,661,674	-	16,685,149	12,297,418	14,004,694	-
LW-LC	9,236,781		-	-	1,587,686		-	-
(LW-LC) - LE	7,990,165			-	1,417,504			-
After alignment to tomato genome	912,215				176,462			-
(LW-LC) - LE intersect WN	114,320				32,970 (set #2)			

	Non-redundant sRNAs
**(PW-PC intersect WN)** intersect **[(LW-LC) - LE intersect WN]**<br>(set #1 intersect set #2)	10,505
