# Supplementary material for: Small RNAs from Bemisia tabaci Are Transferred to Solanum lycopersicum Phloem during Feeding
Source: Front Plant Sci. 2016 Nov 24;7:1759. doi: 10.3389/fpls.2016.01759 (PMC5121246; doi:10.3389/fpls.2016.01759)
Supplement: Supplementary file 2 [file Table2.PDF]

Table S2. Primer sequences used for stem-loop PCR.

**Oligo name**	**sequence**
Rv_miRNA_UniL	CCAGTGCAGGGTCCGAGGTA
RT_miR172	GTCGTATCCAGTGCAGGGTCCGAGGTATTCGCACTGGATACGACatgcag
Fw_miR172	gcggcggAGAATCTTGATGATG
RT_Bt_miR-2a	GTCGTATCCAGTGCAGGGTCCGAGGTATTCGCACTGGATACGACcatcaa
Fw_Bt_miR-2a	ggcggTATCACAGCCAGCT
RT_PW_3182	GTCGTATCCAGTGCAGGGTCCGAGGTATTCGCACTGGATACGACaaagga
Fw_PW_3182	cccgggTAGTAGCTAACGACGAT
RT_PW_13120	GTCGTATCCAGTGCAGGGTCCGAGGTATTCGCACTGGATACGACacagag
Fw_PW_13120	ggccggATTGTACTTCATCAGGTG
RT_PW_18833	GTCGTATCCAGTGCAGGGTCCGAGGTATTCGCACTGGATACGACtaagat
Fw_PW_18833	ggccgTGAGATTCAACTCCTCC
RT_PW_29691	GTCGTATCCAGTGCAGGGTCCGAGGTATTCGCACTGGATACGACaatact
Fw_PW_29691	ggccggggTGCAGGGATATAGTATATATATT
RT_PW_15359	GTCGTATCCAGTGCAGGGTCCGAGGTATTCGCACTGGATACGACtgcgtt
Fw_PW_15359	ggccgggTCCATCAACCAATTGTATAATTTG
RT_PW_23046	GTCGTATCCAGTGCAGGGTCCGAGGTATTCGCACTGGATACGACgaatgc
Fw_PW_23046	gggcgTCTGGAAGATTTGGAATCTTTTGA
