# Supplementary material for: Small RNAs from Bemisia tabaci Are Transferred to Solanum lycopersicum Phloem during Feeding
Source: Front Plant Sci. 2016 Nov 24;7:1759. doi: 10.3389/fpls.2016.01759 (PMC5121246; doi:10.3389/fpls.2016.01759)
Supplement: Supplementary file 3 [file Table3.PDF]

Table S3. Abundance (normalized counts) of previously described *Bemisia tabaci* miRNAs (Guo et al., 2013) in whitefy nymph infested samples.

Sequence (5'-3')	name	LW	WN	PW	Sum
UAGGAACUUCAUACCGUGCUCU	miR-276a-3p	13908	1189707	173362	1,376,977
UGAACACAGCUGGUGGUAUCU	miR-317	2887	181996	14159	199,042
UCAGUCUUUUUCUCUCUCCUAU	miR-14	2788	158481	20052	181,321
UAUCACAGCCACUUUGAUGAACU	miR-2b	2073	137614	11781	151,469
UAGGAACUUCAUACCGUGCUC	miR-276	1593	115382	15817	132,792
UGACUAGAGUUACACUCGUCA	miR-996	2647	85893	3135	91,675
UGAGAUCAUCGUGAAAGCUGAU	bantam	992	59251	5382	65,625
GUGAGCAACGUAUCAGGUGUCU	miR-87a	448	37828	7648	45,925
UGGAAUGUAAAGAAGUAUGGAG	miR-1-3p	900	34700	7946	43,547
UGAGAUCAUCGUGAAAGCUGA	miR-81	620	37974	2101	40,695
UCAGUCUUUUUCUCUCUCCUA	miR-14-3p	661	36826	3453	40,940
UAUCACAGCCACUUUGAUGAAC	miR-13a-3p	640	34329	3570	38,539
UAGCACCAUUUGAAAUCAGUGC	miR-285-3p	846	20667	348	21,861
UCUUUGGUUACCUAGCUGUAUG	miR-9b	265	21114	819	22,198
GUGAGCAAAGUUUCAGGUGUGU	miR-87	309	18670	4681	23,661
UGACUAGAUUUUCACUCAUUCA	miR-279b	347	18736	621	19,704
UGAGUAUUACAUCAGAUAUUUG	miR-12-5p	418	18225	478	19,121
AUUGUACUUCAUCAGGUGCUCUGG	miR-305	90	15650	267	16,008
UGAAAGACAUGGGUAGUGAGA	miR-71c	266	15759	3115	19,141
UAGCACCAUGGAAUUCAGCUU	miR-998	407	15080	1028	16,515
UCCCUGAGACCCUAAUUUGUGA	miR-125b-5p	61	11900	5811	17,772
UAUCACAGCCAGCUUUGAUG	miR-2a	308	13270	1229	14,807
UGGACGGAGAACUGAUAAGGG	miR-184	264	11841	2933	15,038
UAUCACAGCCAUUUUUGACGUG	miR-13b	324	10274	192	10,790
UGAAAGACAUGGGUAGUGAGAUG	miR-71	153	9551	1283	10,987
ACGUAUACUAAAUGUAUCCUGA	miR-iab-4	148	9068	484	9,700
UGGCAGUGUGGUUAGCUGGUU	miR-34	234	9288	878	10,400
UGAGGUAGUAGGUUGUAUAGU	let-7-5p	31	8238	4995	13,264
UGAGGUAGUAGGUUGUAUAGUA	let-7	42	7939	2936	10,918
UGGUAACUCCACCACCGUUGGC	miR-2765	124	7615	484	8,223
UCAGGUACCUGAAGUAGCGCG	miR-275	109	7483	1008	8,600
UCACAGCCAGCUUUGAUGAG	miR-2a-3p	136	7606	936	8,677
UCUCACUACCUUGUCUUUCAUG	miR-71-3p	174	6598	565	7,337
UGGACGGAGAACUGAUAAGGGC	miR-184-3p	109	6337	1254	7,701
UGGCAGUGUGGUUAGCUGGUUGUG	miR-34-5p	89	5675	438	6,202
UAGCACCAUUUGAAAUCAGUG	miR-29b	158	6456	189	6,803
AAUGGCACUAGAAGAAUUCACG	miR-228	113	5560	615	6,288
UCUUUGGUAUUCUAGCUGUAGG	miR-9c-5p	74	5477	379	5,930
UAUUGCACUUGUCCCGGCCUAU	miR-92a	109	4635	745	5,489
UCAGGUACCUGAAGUAGCGCGC	miR-275-3p	38	4155	602	4,794
UUUAGAAUGCUUACGCUUUACC	miR-927-5p	23	4024	490	4,537
AAGAGAGCUAUCCUUCGACAGU	miR-281-2-5p	63	3892	217	4,173
AAUUGCACUAGUCCCGGCCUGC	miR-92b-3p	90	3476	571	4,137
UGGAAGACUAGUGAUUUUGUUGU	miR-7-5p	51	3429	249	3,730
UAAAUGCACUAUCUGGUACGACA	miR-277-3p	36	2995	1781	4,812
UCGUCAAAUGGUUGUGAAGUG	miR-2b-2-5p	44	3619	371	4,033
UCAGGUACUGAGUGACUCUGA	miR-306	114	3541	85	3,740
AGGCAAGAUGUUGGCAUAGCUGA	miR-72	36	2952	184	3,172
UACCCUGUAGAUCCGGGCUUUUG	miR-993-5p	62	2908	107	3,077
AACCCGUAGAUCCGAACUUGU	miR-100	11	3025	1008	4,045
UGUCAUGGAGUUGCUCUCUUUG	miR-281	101	2962	137	3,199
UAUUGCUUGAGAAUACACGUAG	miR-137-3p	69	2827	261	3,156
AUAUUGUCCUGUCACAGCAGUA	miR-1000	32	2599	236	2,868
UCUCACUACCUUGUCUUUCAU	miR-71*	44	2519	241	2,803
UUGGUCCCCUUCAACCAGCUGU	miR-133-3p	17	2273	584	2,874
UGGAAGACUAGUGAUUUUGUU	miR-7	61	2366	150	2,576
UGUCUUUUCCCGCUUUGCUGC	miR-316-5p	49	2227	442	2,719
CCAGAUCUAACUCUUCCAGCUCU	miR-750	255	1788	249	2,293
AGGCAAGAUGUUGGCAUAGCUG	miR-31	15	1768	130	1,913
AAUGGCACUAGAAGAAUUCACGGG	miR-263a-5p	37	1406	245	1,687
UGACUAGAUUUUCACUCAUUC	miR-279d	45	1631	26	1,702
GAAGCUCGUCUCUACAGGUAUC	miR-993-3p	39	1533	130	1,703
UCAUCAAAAUGGUUGUGGAAUG	miR-2a-1-5p	27	1526	74	1,628
UUUGUUCGUUCGGCUCGAGUUA	miR-375	12	1414	230	1,656
UCACAGCCAGCUUUGAUGAGU	miR-2c	28	1329	124	1,481
UAAAUGCACUAUCUGGUACGAC	miR-277	13	1206	397	1,617
UAGCACCAUGGAAUUCAGCUUU	miR-285	28	1241	112	1,381
UGUCUUUUCCCGCUUUGCUGU	miR-316*	28	1285	189	1,501
AAUGGCACUAGAAGAAUUCAC	miR-263a	39	1245	228	1,512
UGAGAUUCAACUCCUCCAUCUU	miR-1175-3p	22	1175	68	1,265
UAAGGCACGCGGUGAAUGCCAUU	miR-124-3p	13	1105	166	1,284
UCACAACCUCCUUGAGUGAGC	miR-307	14	1146	78	1,238
CUACCCUGUAGAUCCGGGCUUUUG	miR-993*	21	838	34	893
UACCCUGUAGAUCCGGGCUUUU	miR-993a*	44	872	19	935
UAAUACUGUCAGGUAAUGAUG	miR-8	14	900	NA	914
UUAUUGCUUGAGAAUACACGU	miR-137	4	876	104	984
CUAAGUACUCCGUGCCGCAGGA	miR-252a	13	778	186	977
UUGGUCCCCUUCAACCAGCUG	miR-133	14	749	189	952
GUAGGCCGGCGGAAACUACUUGC	miR-2796-3p	12	664	232	908
GUGAGCAAAGUUUCAGGUGUG	miR-87a-3p	30	694	104	828
UACCCUGUAGAUCCGGGCUUU	miR-993b*	31	587	13	631
UACGUAUACUAAAGGUAUACCGG	miR-iab-5p	9	550	12	571
UUAUUGCUUGAGAAUACACGUAG	miR-137b	15	506	48	569
UGUCAUGGAGUUGCUCUCUUU	miR-281-3p	24	517	26	567
AGAUAUGUUUGAUAUUCUUGG	miR-190-5p	8	485	65	559
UAAUACUGUCAGGUAAUGAUGUU	miR-200b	9	423	12	444
UGUCUUUUCCCGCUUUGCUGCC	miR-316	7	393	112	512
AGCGAGGUAUAGAGUUCCUACGU	miR-276-5p	3	368	12	382
UCGGUGGGACUUUCAUCUGA	miR-278	1	360	7	369
AUAUUGUCCUGUCACAGCAG	miR-1000-5p	1	340	14	355
UUGUGCGUGUGACAGCGGCU	miR-210	3	287	150	440
UUGUGCGUGUGACAGCGGCUA	miR-210-3p	NA	256	228	484
ACCCUGUAGAUCCGAAAUUUGU	miR-10	4	280	43	327
GUGAGCAAAGUUUCAGGUGUGUU	miR-87b-3p	4	258	77	339
CUUGGCACUGGAAGAAUUCACAGA	miR-263b	4	247	28	279
UCGUCAAAUGGUUGUGAAGU	miR-13a-5p	1	250	20	272
AUUGUACUUCAUCAGGUGCUCUG	miR-305-5p	3	210	NA	213
UGAACACAGCUGGUGGUAUCA	miR-317-3p	1	229	7	237
UUGGUGUUCUACCUUACAGUG	miR-971	14	206	7	227
UUGGUCCCCUUCAACCAGCU	miR-133b	1	223	20	245
CAAAGCGUUUGAAUUCUGGAAC	miR-927-3p	NA	202	6	209
UCCGUCCAACUCUUUUCCGCCU	miR-3049	4	181	81	265
UUUUGAUUGUUGCUCAGAAAGCC	miR-315	3	159	NA	161
AAAUUGACUCUAGUAGGGAGU	miR-929-5p	1	174	NA	175
AAAUUGACUCUAGUAGGGAG	miR-929	4	160	41	206
GUAGGCCGGCGGAAACUACU	miR-2796	3	163	27	194
UUUUGAUUGUUGCUCAGAAAGC	miR-315-5p	3	154	NA	157
UGAGUAUUACAUCAGAUAUUUGA	miR-12	12	114	NA	126
AUCUUACUGGGCAGCAUUGGA	miR-200b*	4	121	13	138
CUAAGUAGUAGCGCCGAAGGU	miR-252	3	110	7	119
UGGACGGAGAACUGAUAAGGA	miR-184b	NA	104	26	130
UUGGUCCCCUUCAACCAGCUGC	miR-133c	NA	87	43	131
CUUGGCACUGGAAGAAUUCAC	miR-263b-5p	NA	95	7	101
UUCGUUGUCGCCGAAAACUCG	miR-981	NA	76	46	122
AUAAAGCUGGAUUACCAAAUCG	miR-9d-3p	NA	63	81	143
UCACAACCUCCUUGAGUGAG	miR-307a-3p	3	80	14	97
AGCUGCUGGACACUGCACAAGA	miR-210*	NA	58	43	102
AUAAAGCUGGAUUACCAAAGCG	miR-9b-3p	NA	41	31	72
CAAAUUCGGUUCUAGAGAGGU	miR-10-3p	NA	43	26	69
UAUUGCACUUGUCCCGGCCUU	miR-92b	1	46	NA	47
UAAGCGUAUAGCUUUUCCCCU	miR-965-3p	1	34	20	54
UGACUAGAGAUACACUCGCU	miR-279e	6	29	NA	35
CUAAGUAGUAGCGCCGAAGGUGA	miR-252b	NA	26	12	38
AGAUAUGUUUGAUAUUCUUGGU	miR-190	NA	23	12	36
UCGGGAAGGCCGUUGCGGCGGA	miR-3049-5p	NA	25	6	31
UCAUAUCCGGCUCGGAGGACCA	miR-2779	NA	22	NA	22
UGAGAUCAUCGCGAAAGCUGAU	bantam-b	NA	19	NA	19
AAUUGCACUAGUCCCGGCCUA	miR-92a-3p	NA	17	NA	17
UCAGAUCUAACUCUUCCAGCUCU	miR-750-3p	NA	15	NA	15
AUAAAGCUGGAUUACCAAAGCGU	miR-79	NA	11	12	23
UAAGUAGUAGCGCCGAAGGU	miR-252b-5p	NA	14	NA	14
UACCCUGUAGAUCCGAAAUUUG	miR-10a	NA	13	NA	13
UGAGAUCAUCAUGAAAGCUGAU	bantam-3p	NA	13	NA	13
UCUUUGGUUACCUAGCUGUGUG	miR-9	NA	12	NA	12
AACCCGUAGAUCCGAACUUGUGAA	miR-100-5p	NA	7	6	12
UAGCACCAUUUGAAAUCAGA	miR-29a	NA	8	NA	8
UGAGAUCAUCAUGAAAGCUGA	bantam*	NA	6	NA	6
UGAGAUCAUCGUGAAAGCCGA	miR-82	NA	6	NA	6
UGAGGUAGUAGAUUGUAUAGU	let-7f	NA	5	7	11
CCCCUGAGACCCUAAUUUGUGA	miR-125	NA	4	NA	4
AAAUUCGGUUCUAGAGAGGUU	miR-10*	NA	3	NA	3
CUUGGCACUGGAAGAAUUCACU	miR-182	NA	3	NA	3
UGGAAUGUAAAGAAGUAUGGUU	miR-1b	NA	3	NA	3
AGCUGGUUGAACCUGGGUCA	miR-133-5p	NA	2	NA	2
AUUGCACUAGUCCCGGCCUGCU	miR-92c	NA	1	NA	1
CUUGUUCGUUCGGCUCGAGU	miR-375-3p	NA	2	NA	2
UAAUACUGCCAGGUAAUGAUG	miR-200c	NA	2	NA	2
UAUCAAUAGACAAAGGCUCGUAGC	bta-miRn2	NA	1	NA	1
UCUGAAACAGAGAGGGAUAUCGUG	bta-miRn15	NA	NA	6	6
UCUUUGGUAUUCUAGCUGUAGGAU	miR-9a	NA	1	NA	1
UGGAAUGUAAGGAAGUAUGG	miR-206	NA	2	NA	2
UAAUACUGUCAGGUAAAGAUGUC	miR-8-3p	NA	NA	NA	-
