# Supplementary material for: Small RNAs from Bemisia tabaci Are Transferred to Solanum lycopersicum Phloem during Feeding
Source: Front Plant Sci. 2016 Nov 24;7:1759. doi: 10.3389/fpls.2016.01759 (PMC5121246; doi:10.3389/fpls.2016.01759)
Supplement: Supplementary file 4 [file Table4.PDF]

Table S4. Abundance (normalized counts) of detectable *Solanum lycopersicum* miRNAs (from miRBase v21) in non-infested samples.

miRNA	LC (Leaf control)	PC (Phloem control)
sly-miR156a	1333	1703
sly-miR156b	1140	1915
sly-miR156c	1199	1782
sly-MIR156d	114	NA
sly-MIR156e	83	NA
sly-miR156e-3p	NA	372
sly-miR159	9101	36657
sly-MIR160a	10	NA
sly-miR162	4344	21743
sly-MIR164a	202	NA
sly-MIR164b	74	NA
sly-MIR166a	19	NA
sly-MIR166b	26	NA
sly-MIR166c	55	NA
sly-miR167a	2798	2634
sly-MIR167b	3079	NA
sly-MIR168a	1969	NA
sly-miR168a-5p	NA	434
sly-MIR168b	1409	NA
sly-miR168b-5p	NA	568
sly-MIR169a	30	NA
sly-MIR169b	25	NA
sly-MIR169c	36	NA
sly-MIR169d	49	NA
sly-MIR169e	64	NA
sly-miR169e-3p	NA	1224
sly-miR171a	406	5241
sly-MIR171b	59	NA
sly-MIR171c	2151	NA
sly-MIR171d	50	NA
sly-miR171e	3321	8637
sly-miR172a	14686	7493
sly-miR172b	1868	6757
sly-MIR1916	2700	NA
sly-MIR1917	8321	NA
sly-MIR1918	456	NA
sly-MIR1919a	491	NA
sly-MIR1919b	897	NA
sly-MIR1919c	917	NA
sly-miR1919c-5p	NA	621
sly-MIR319a	13	NA
sly-MIR319b	166	NA
sly-MIR319c	56	NA
sly-MIR390a	658	NA
sly-MIR390b	136	NA
sly-miR390b-5p	NA	284
sly-MIR394	674	NA
sly-miR394-5p	NA	275
sly-MIR395a	47	NA
sly-MIR395b	2	NA
sly-MIR396a	13003	NA
sly-miR396a-3p	NA	1454
sly-miR396a-5p	NA	6526
sly-miR396b	7115	12796
sly-MIR397	6599	NA
sly-MIR399	49	NA
sly-MIR403	44536	NA
sly-miR403-3p	NA	3902
sly-miR4376	13314	638
sly-MIR477	257	NA
sly-miR482a	906	3166
sly-miR482b	30415	22567
sly-miR482c	3537	5374
sly-MIR482d	3494	NA
sly-miR482d-3p	NA	1871
sly-MIR482e	191649	NA
sly-miR482e-3p	NA	180157
sly-miR482e-5p	NA	3760
sly-miR5300	15024	2509
sly-MIR5302a	54	NA
sly-MIR5302b	55	NA
sly-MIR5303	319	NA
sly-miR5304	1408	275
sly-miR6022	69008	10339
sly-MIR6023	19445	NA
sly-MIR6024	13774	NA
sly-MIR6026	2359	NA
sly-MIR6027	8858	NA
sly-miR6027-3p	NA	2323
sly-MIR9469	448	NA
sly-MIR9470	4784	NA
sly-MIR9471a	4922	NA
sly-miR9471a-3p	NA	771
sly-MIR9471b	5117	NA
sly-miR9471b-3p	NA	532
sly-MIR9472	529	NA
sly-MIR9473	140	NA
sly-MIR9474	2850	NA
sly-MIR9475	1202	NA
sly-MIR9476	416	NA
sly-MIR9477	247	NA
sly-MIR9478	1430	NA
sly-MIR9479	317	NA
sly-miR9479-3p	NA	301
sly-miR9479-5p	NA	310
