# Supplementary figures and images for: Small RNAs from Bemisia tabaci Are Transferred to Solanum lycopersicum Phloem during Feeding
Source: Front Plant Sci. 2016 Nov 24;7:1759. doi: 10.3389/fpls.2016.01759 (PMC5121246; doi:10.3389/fpls.2016.01759)

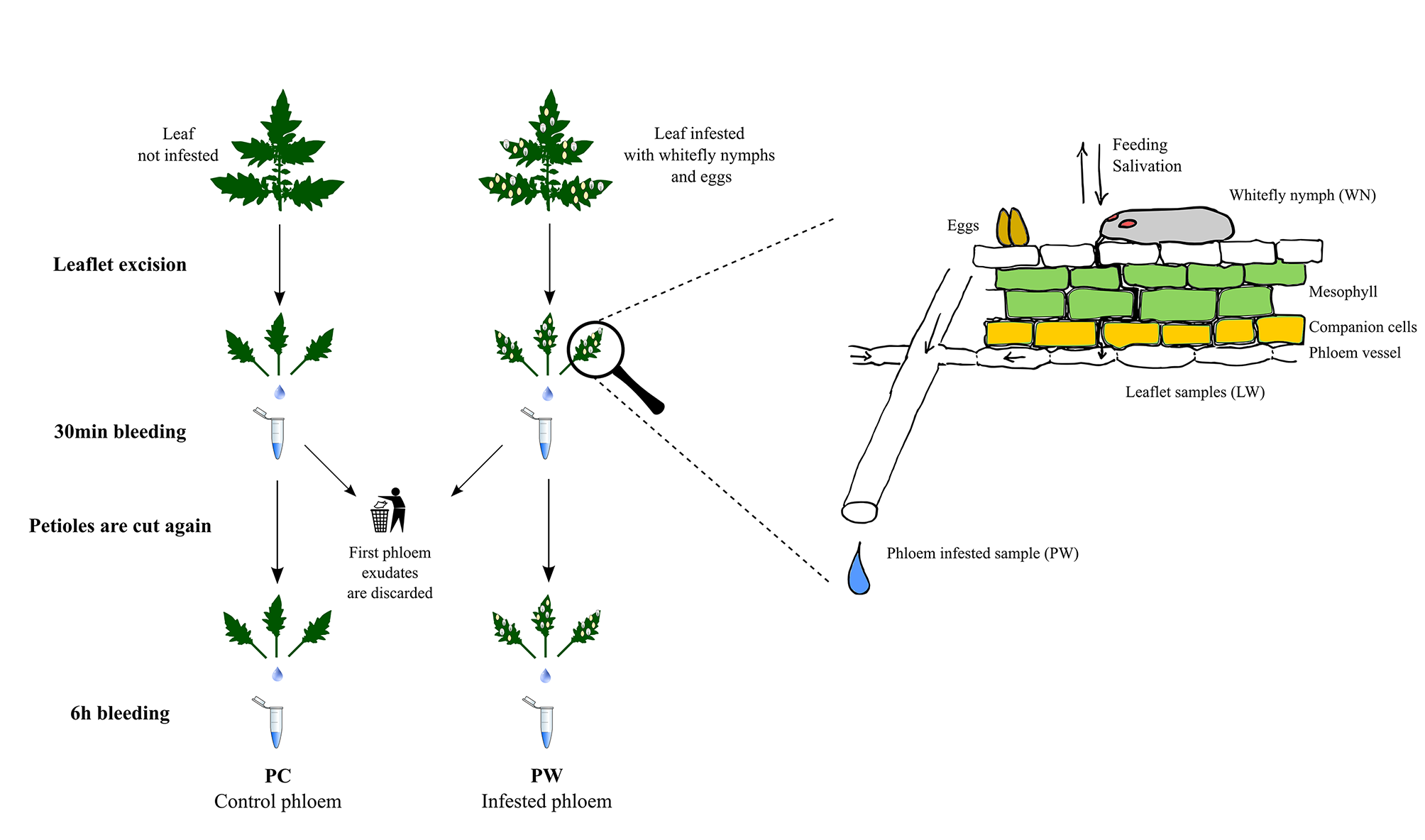

Supplement: Supplementary file 5 [file Image1.TIF]
